# Efficacy and Safety of Exercise Rehabilitation for Heart Failure Patients With Cardiac Resynchronization Therapy: A Systematic Review and Meta-Analysis

**DOI:** 10.3389/fphys.2020.00980

**Published:** 2020-08-21

**Authors:** Li-fang Ye, Shao-mei Wang, Li-hong Wang

**Affiliations:** Department of Cardiovascular Medicine, Zhejiang Provincial People's Hospital, People's Hospital of Hangzhou Medical College, Hangzhou, China

**Keywords:** cardiac rehabilitation, heart failure, cardiac resynchronization therapy, meta-analysis, randomized controlled trial

## Abstract

**Background:** Many heart failure (HF) patients admitted to cardiac rehabilitation (CR) centers have a cardiac resynchronization therapy (CRT) device. However, information about the efficacy and safety of exercise rehabilitation in HF patients with a CRT device is scant. We assessed the effects of exercise rehabilitation in HF patients with a CRT device.

**Methods and Results:** The PubMed, EMBASE, Cochrane Central Register of Controlled Trials, CINAHL, PsycInfo, China Biology Medicine, Wanfang, and China National Knowledge Infrastructure databases were searched comprehensively to identify randomized controlled trials (RCTs) published between January 1, 1990 and July 1, 2019 on exercise rehabilitation in HF patients with CRT devices. We identified seven studies published from 2006 to 2019, including 661 patients with an intervention duration of 8 to 24 weeks. Three studies reported all-cause mortality and serious adverse events, and no significant difference was found between exercise rehabilitation patients and controls at the longest available follow-up (both *P* > 0.05; both *I*^2^ = 0%). Exercise rehabilitation patients exhibited a higher exercise capacity (peak oxygen uptake: random-effect WMD = 2.02 ml/kg/min, 95% CI 0.62 to 3.41, *P* = 0.005, *I*^2^ = 67.4%; exercise duration: fixed-effect WMD = 102.34s, 95% CI 67.06 to 137.62, *P* < 0.001, *I*^2^ = 25%) after intervention, despite the significant heterogeneity of studies. Left ventricular ejection fraction (LVEF) was significantly improved in exercise rehabilitation patients compared to that in controls (fixed-effect WMD = 3.89%, 95% CI 1.50 to 6.28; *P* = 0.001; *I*^2^ = 48.0%). Due to differences in health-related quality of life (HRQOL) assessment methods, we only pooled data that reported Minnesota Living with Heart Failure Questionnaire scores. Exercise rehabilitation patients exhibited a better HRQOL than controls (fixed-effect WMD = −5.34, 95% CI −10.12 to −0.56; *P* = 0.028; *I*^2^ = 0%).

**Conclusions:** Exercise rehabilitation may restore exercise capacity and cardiac function in HF patients with a CRT device. Furthermore, exercise training was associated with better HRQOL on follow-up.

## Introduction

Heart failure (HF) is the end stage of most cardiovascular diseases, and is a leading cause of death (Normand et al., 2019). Although the prognosis of patients with HF has improved with evidence-based treatment, patients eventually progress to the advanced stages of the disease (Crespo-Leiro et al., 2018). The treatment and management of HF remains a significant public health burden in the United States, with millions of patients suffering from the disease, many of them facing a poor prognosis (Brown et al., 2018).

Cardiac resynchronization therapy (CRT) is currently a well-established treatment for HF patients with severe left ventricular systolic dysfunction and ventricular systolic dyssynchrony (Bristow et al., 2004; Cleland et al., 2005). A number of clinical randomized controlled trials (RCTs) have shown that a CRT device can improve heart function and structure, health-related quality of life (HRQOL), and exercise capacity and reduce hospitalization and mortality in progressive HF patients (Duncan et al., 2003; Cleland et al., 2006; Tang et al., 2010). However, approximately 20–30% of patients show little or no response (Abraham et al., 2002).

Cardiac rehabilitation (CR) is a widely accepted treatment strategy for chronic HF patients and has been proven to reduce mortality and improve exercise capacity, heart function, HRQOL, and prognosis (Piepoli et al., 2004; van Tol et al., 2006). However, most published studies focus on the effect of exercise rehabilitation on New York Heart Association (NYHA) I-II patients with HF, with little research paying attention to NYHA II-IV HF patients. HF patients with indications of CRT implantation tend to have a higher level of NYHA functional class (Yancy et al., 2013). Some experts have proposed that CRT is usually programmed at rest, and an exercise test may detect a loss of resynchronization during exercise training, which may weaken the benefits of CRT (Iliou et al., 2016). However, several studies also reported that exercise rehabilitation might result in a better response to CRT in HF patients (Conraads et al., 2007). Therefore, their combined effect on HF patients is controversial. Furthermore, information about the safety and efficacy of exercise rehabilitation in HF patients with a CRT device is scarce.

With the rapidly growing rate of CRT implantations in patients with HF (Piccini et al., 2013), a relatively large proportion of patients admitted to CR centers have a CRT device. Consequently, much more attention should be paid to exercise rehabilitation in HF patients with a CRT device. Recently, Chen et al. published a meta-analysis to evaluate the effects of exercise rehabilitation on peak oxygen uptake (peak VO_2_) and left ventricular ejection fraction (LVEF) in HF patients with a CRT device. However, this study failed to perform a systematic and comprehensive analysis on the safety and other efficacy end points of exercise rehabilitation in HF patients with a CRT device, such as all-cause mortality, serious adverse events and HRQOL, which are the most significant prognostic indicators in HF patients (Chen et al., 2019). Accordingly, we conducted a systematic review and meta-analysis to assess exercise rehabilitation in HF patients with a CRT device and to determine study design, safety, and effectiveness of exercise training in these patients.

## Methods

This study protocol has been published previously in PROSPERO (CRD42020162738).

### Literature Search Strategy

The PubMed, EMBASE, Cochrane Central Register of Controlled Trials, CINAHL, PsycInfo, China Biology Medicine, Wanfang, and China National Knowledge Infrastructure databases were searched comprehensively to identify RCTs published between January 1, 1990 and July 1, 2019. The keywords for this search included: (exercise or training or rehabilitation) and (biventricular or resynchronization) and heart failure. We also scanned the references in the included studies for relevant results. The detail search strategy for PubMed is documented in Supplementary Table 1.

### Literature Selection Criteria

Two authors (SW and LY) independently screened all the included studies. This meta-analysis was planned following the PRISMA guidelines (Moher et al., 2009). The following studies were included: (1) RCTs, (2) those involving HF patients (>18 years of age) with a CRT device, (3) those comparing exercise rehabilitation and controls (HF patients with and without a CRT device) (exercise rehabilitation was defined as an intervention that included exercise training, either alone or in addition to psychosocial and/or educational interventions), (4) those that included of a control group that received standard medical care without exercise training or advice, (5) those with data on at least one of the following outcome measurements: echocardiographic measures (LVEF, left ventricular end diastolic dimension [LVEDD]), exercise capacity (peak VO_2_, exercise duration), HRQOL, and/or adverse events (all-cause mortality, serious adverse events). The following studies were excluded: (1) those with incomplete data, (2) duplicate reports, (3) case reports, reviews, or animal studies, (4) those with interventions that did not include exercise training, and (5) those with participants without a CRT device.

### Data Extraction

Two authors (SW and LY) independently extracted the relevant data from eligible articles using a predesigned data extraction form. The article titles and abstracts were first screened to identify potentially eligible studies and then the full paper was reviewed. Any disagreements were resolved through discussion. The extracted information included the first author, publication year, sample size, exercise training duration, follow-up time, echocardiographic measures (LVEF, LVEDD), exercise capacity (peak VO_2_, exercise duration), HRQOL, adverse events (all-cause mortality, serious adverse events), and exercise rehabilitation protocol. Serious adverse events were defined as life-threatening events or those leading to death, hospitalization, or permanent or significant disability (Nielsen et al., 2019).

### Study Evaluation and Risk of Bias Assessment

We used the Cochrane Collaboration's tool to assess the methodological quality of all included studies (Higgins and Green, 2011). Additionally, baseline imbalance was evaluated. Two authors (SW and LY) independently evaluated bias assessment. Any disagreements were resolved by discussion.

### Statistical Analysis

We used the STATA software package, version 12.0 (Stata Corporation, College Station, TX) to perform all statistical analyses. The weighted mean difference (WMD) with 95% confidence interval (CI) between exercise rehabilitation and the control group was calculated to estimate the pooled effects. Heterogeneity was assessed using Cochrane's *Q*-test and *I*^2^ statistics. *I*^2^-values of 25, 50, and 75% indicated low, moderate, and high heterogeneity, respectively. When *P* > 0.05 or *I*^2^ < 50%, the heterogeneity was not significant, and a fixed-effect model was used. Otherwise, it was considered to indicate a statistical heterogeneity among the included studies, and the random-effect model was adopted. We performed sensitivity analysis by omitting each study in turn from the pooled analysis. Publication bias was evaluated by Begg's funnel plot and Egger's regression test. *P* < 0.05 was considered statistically significant.

## Results

### Identification and Selection of Studies

**Figure 1:**
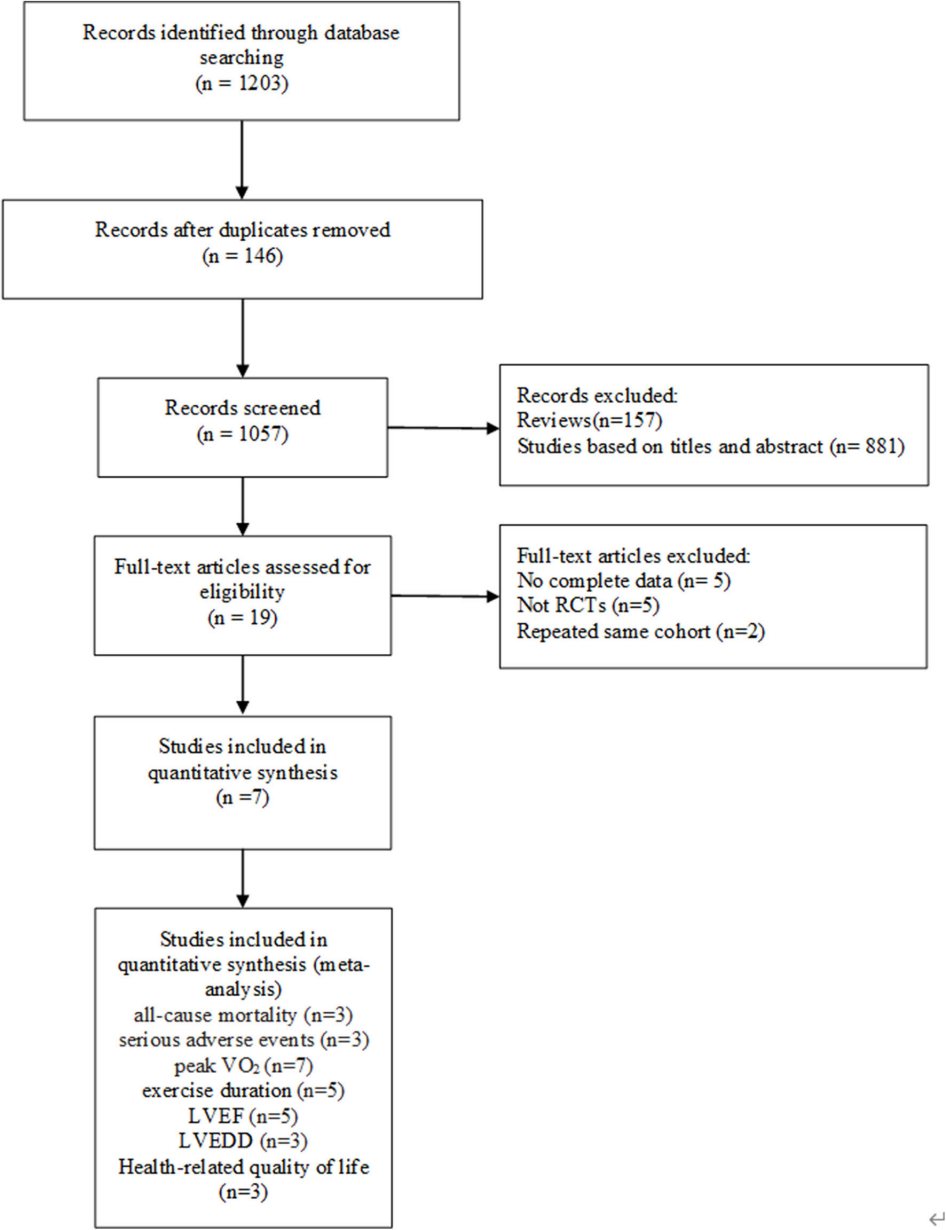
Flow diagram of the study selection procedure.

A preliminary search of the literature yielded 1,203 articles. One hundred and forty-six papers were duplicates, 157 papers were reviews, and 881 papers were excluded based on titles and abstracts. The full text of the remaining articles was retrieved and evaluated according to the inclusion criteria. The reasons for the exclusion of 12 articles were as follows: five articles provided incomplete data, five articles were designed as non-RCTs, and two articles reported the same cohort. In the end, seven studies (Belardinelli et al., 2006; Conraads et al., 2007; Patwala et al., 2009; Smolis-Bak et al., 2015; Zeitler et al., 2015; Nobre et al., 2016; Santa-Clara et al., 2019) were identified for analysis. The PRISMA flow chart for this meta-analysis is presented in Figure 1. The characteristics of the studies and participants are shown in Tables 1, 2.

**Table 1:** Design and interventions of included studies.

**References**	**Follow-up time(month)**	**Interval between CRT and ER(week)**	**Intervention**	**Patient Population**	**Location**	**Outcomes**
Belardinelli et al. (2006)	24 ± 6	12	Supervised exercise training for 1 h, 3 times/week for 8 weeks at 60% peak VO_2_, each session consisted of 15 min stretching, 40 min cycling, and 5 min loadless recovery	52 men in NYHA class II and class III with ischemic cardiomyopathy, 30 intervention (15 ICD, 15 CRT-D) and 22 control patients (12 ICD, 10 CRT-D)	Italy and United States	CPET, brachial artery vasomotor function, QOL, hospital readmission, echocardiographic measures, NYHA class, ICD shocks
Conraads et al. (2007)	5	4	Supervised exercise training for 1 h, 3 times/week for 4 months at 90% of the ventilatory threshold during CPET, each session consisted of 5 min warming up and stretching, followed by endurance training (cycling, walking) and 5 min cooling down.	17 patients receiving CRT (13 CRT-P, 4 CRT-D), ischaemic or dilated cardiomyopathy with NYHA class III-IV, 8 intervention and 9 control patients; matched historical HF cohort, 9 intervention and 10 control patients	Belgium	CPET, QOL, echocardiographic measures, NT-proBNP, intraventricular delay
Patwala et al. (2009)	6	12	Supervised exercise training for 30 min, 3 times/week for 3 months at 80–90% of the peak heart rate, each session consisted of 10 min treadmill walking, 10 min cycling, and 10 min treadmill walking	50 patients in NYHA functional class III to IV undergoing new CRT, 25 intervention and 25 control patients	United Kingdom	CPET, QOL, echocardiographic measures, NYHA class
Smolis-Bak et al. (2015)	18	NA	Exercise training in hospital rehabilitation unit for 3 weeks on average with home telemonitored training 5 times/week for 8 weeks. Each session consisted of exercises of small and larger muscle groups, respiratory exercises	52 patients in NYHA functional class III receiving CRT, 26 intervention and 26 control patients	Poland	CPET, QOL, echocardiographic measures, ICD shocks
Zeitler et al. (2015) (*post hoc* analysis)	3	At least 6	Supervised exercise training for 15–30 min, 3 times weekly for 6 sessions, increased to 30–35 min, 3 times/week for 30 sessions, with 40 min home-based exercise 5 times/week after completing 18 supervised sessions; treadmill walking and cycling	1,118 patients with NYHA class II-IV, LVEF ≤35%, who had ICDs, 575 intervention (351 RV, 224 BiV) and 543 control patients (332 RV, 211 BiV)	United States	CPET, QOL, echocardiographic measures, ICD shocks
Nobre et al. (2016)	4	4	Supervised exercise training for 1 h, 3 times/week for 4 months, each session consisted of 5 min stretching, 40 min treadmill walking, 10 min strengthening, and 5 min stretching	45 patients in NYHA functional class I to III receiving CRT, 23 intervention and 22 control patients	Brazil	CPET, muscle sympathetic nerve activity, echocardiographic measures, forearm blood flow, expression in vastus lateralis muscle, Ca2+ handling gene
Santa-Clara et al. (2019)	6	2–4	Supervised exercise training program for 1 h, 2 times/week for 6 months, each session consisted of 4 high intensity interval training periods with 3 moderate intensity active periods between interval training periods	63 patients in NYHA functional class II to IV receiving CRT, 34 intervention and 29 control patients		CPET, QOL, echocardiographic measures, NYHA class

*ER, exercise rehabilitation; CRT-D, cardiac resynchronization therapy defibrillator; ICD, implantable cardioverter-defibrillator; NYHA, New York Heart Association; CPET, cardiopulmonary exercise testing; QOL, quality of life*.

**Table 2:** Clinical characteristics of the study population included in the meta-analysis.

	**Belardinelli et al**.	**Conraads et al**.	**Patwala et al**.	**Smolis-Bak et al**.	**Zeitler et al**.	**Nobre et al**.	**Santa-Clara et al**.
Con/ER	10/15	9/8	25/25	26/26	211/224	22/23	29/34
% Males	NA	47.1	92	90.4	78.4	53.3	75.7
Age, years	NA	59.1	64.4	62.6	61	54.6	67.5
BMI, kg/m^2^	NA	NA	NA	28.4	30	26.8	27.4
% Atrial fibrillation	NA	0	34	61.5	4.1	0	51.4
% NYHA functional class II	NA	0	0	0	52.6	53.3	NA
% NYHA functional class III or IV	NA	100	100	100	47.4	28.9	NA
% Diabetes	NA	NA	NA	25	33.1	NA	NA
% History of MI	NA	NA	NA	44.2	44.4	NA	NA
% LVEF	NA	27.5	23.7	25.1	23	27.6	26.3
% Heart failure etiology:							
Ischemic	100	23.5	NA	46.2	52.6	13.3	37.8
Other	0	76.5	NA	53.8	47.4	86.7	62.2
% Medications							
Beta-blocker	NA	100	60	100	94	100	87.8
ACE-I/ARB	NA	100	98	73.8	93.1	84.4	90.5
MRA	NA	88.2	54	83.4	55.2	75.6	NA
Digitalis	NA	35.3	46	NA	57.2	28.9	NA
Diuretics	NA	88.2	96	80.3	NA	62.2	92.6
Antiarrhythmics	NA	NA	NA	NA	28.5	NA	NA

*Values are mean or %. BMI, body mass index; Con, control; ER, exercise rehabilitation; CRT, cardiac resynchronization therapy; NYHA, New York Heart Association; LVEF, left ventricular ejection fraction; MI, myocardial Infarction; ACE-I, angiotensin-converting enzyme inhibitor; ARB, angiotensin receptor; MRA, mineralocorticoid receptor antagonist; NA, data not available*.

### Description of Included Trials

We included seven trials in which the median duration of exercise training was 16 weeks (range: 8 to 24 weeks) and the median follow-up of all-cause mortality and serious adverse events was 6 months (range: 2 to 30 months). The interval time between CRT implantation and the start of exercise training ranges from 2 to 12 weeks. At medication use baseline, most participants used beta-blockers and angiotensin-converting enzyme inhibitors/angiotensin-receptor blockers. In contrast, a lower proportion of patients were receiving treatment with mineralocorticoid receptor antagonists. In most included studies, males and participants with NYHA functional class III or IV represented the majority of the study population (Table 2). The detailed exercise rehabilitation program protocols of the included studies are presented in Table 1.

### Outcomes

#### All-Cause Mortality

**Figure 2:**
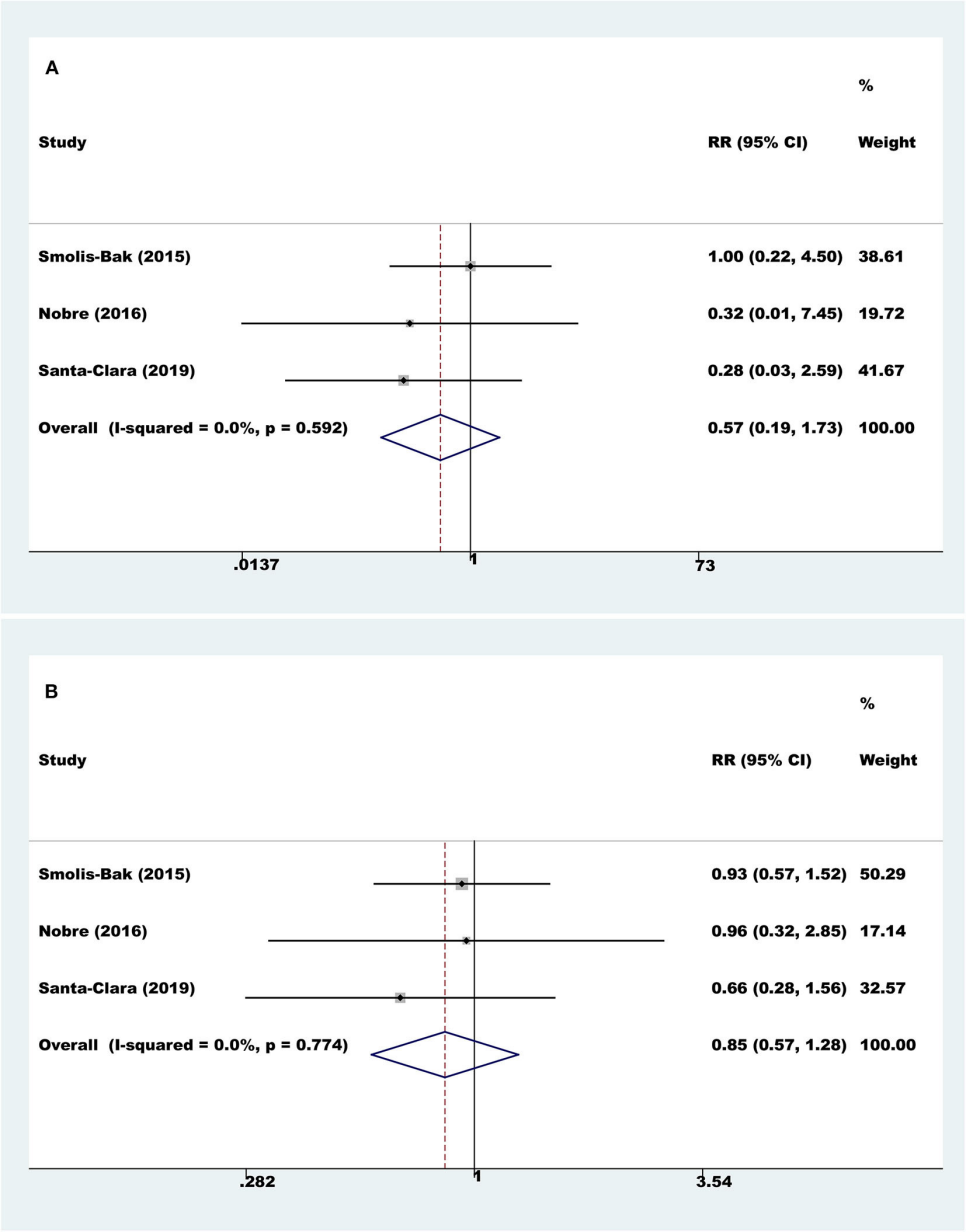
Meta-analyses of all-cause mortality and serious adverse events. **(A)** All-cause mortality; **(B)** serious adverse events.

Three trials with a total of 160 participants and a follow-up ranging from 4 to 18 months reported all-cause mortality. Meta-analysis showed that there was no evidence of a difference between the exercise rehabilitation group and control group at the longest available follow-up (fixed-effect relative risk 0.57, 95% CI 0.19 to 1.73; *P* = 0.32; *I*^2^ = 0%, Figure 2A).

#### Serious Adverse Events

Three trials with a total of 160 participants and a follow-up ranging from 4 to 18 months reported results on serious adverse events. Meta-analysis showed that there was no evidence of a difference between the exercise rehabilitation group and the control group at the longest available follow-up (fixed-effect relative risk 0.85, 95% CI 0.57 to 1.28; *P* = 0.43; *I*^2^ = 0%, Figure 2B).

#### Exercise Capacity

**Figure 3:**
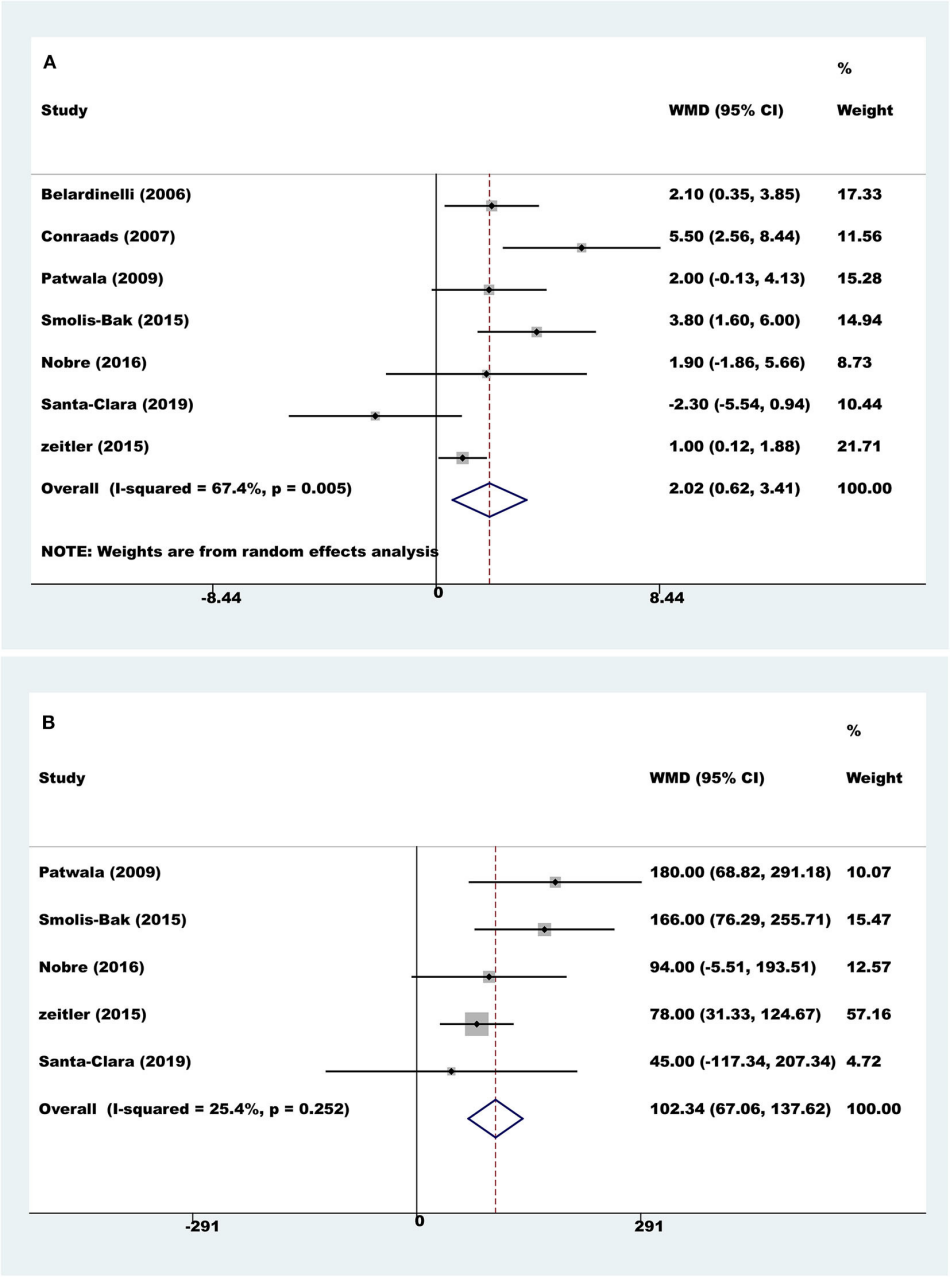
Meta-analyses of exercise capacity. **(A)** Peak oxygen uptake; **(B)** exercise duration.

Seven trials with a total of 560 participants reported results on peak VO_2_. Compared to controls, pooled peak VO_2_ was higher with exercise rehabilitation (random-effect WMD = 2.02 ml/kg/min, 95% CI 0.62 to 3.41; *P* = 0.005; *I*^2^ = 67.4%, Figure 3A). The sources of heterogeneity were determined by the Galbraith plot, and there was significant heterogeneity in two of the studies (Supplementary Image 1). The heterogeneity was remarkably decreased after excluding these two studies, and the differences remained significant (random-effect WMD = 1.88 ml/kg/min, 95% CI 0.88 to 2.89; *P* < 0.001; *I*^2^ = 34.2%). There was no significant change in the pooled effect by sensitivity analysis.

Four trials with a total of 528 participants reported results on exercise duration. Compared to controls, pooled exercise duration was higher with exercise rehabilitation (fixed-effect WMD = 102.34 s, 95% CI 67.06 to 137.62; *P* < 0.001; *I*^2^ = 25%, Figure 3B).

#### Cardiac Function

**Figure 4:**
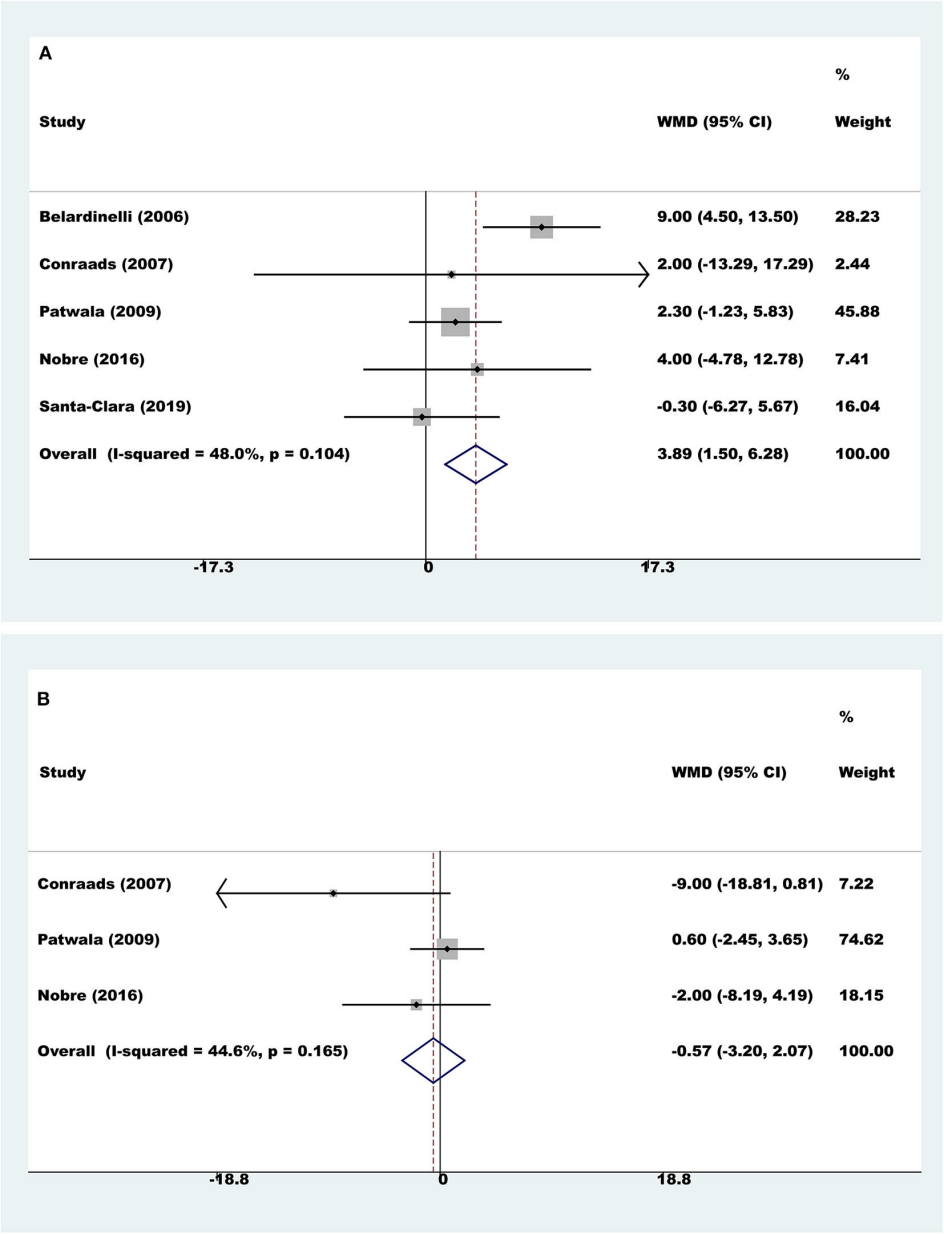
Meta-analyses of cardiac function. **(A)** Left ventricular ejection fraction; **(B)** left ventricular end diastolic dimension.

Five trials with a total of 159 participants reported results on LVEF. Compared to controls, pooled LVEF was higher with exercise rehabilitation (fixed-effect WMD=3.89%, 95% CI 1.50 to 6.28; *P* = 0.001; *I*^2^ = 48.0%, Figure 4A).

Three trials with a total of 97 participants reported results on LVEDD. Meta-analysis showed that there was no evidence of a difference between the exercise rehabilitation group and control group (fixed-effect WMD = −0.57 mm, 95% CI 3.20 to 2.07; *P* = 0.674; *I*^2^ = 44.6%, Figure 4B).

#### Health-Related Quality of Life

**Figure 5:**
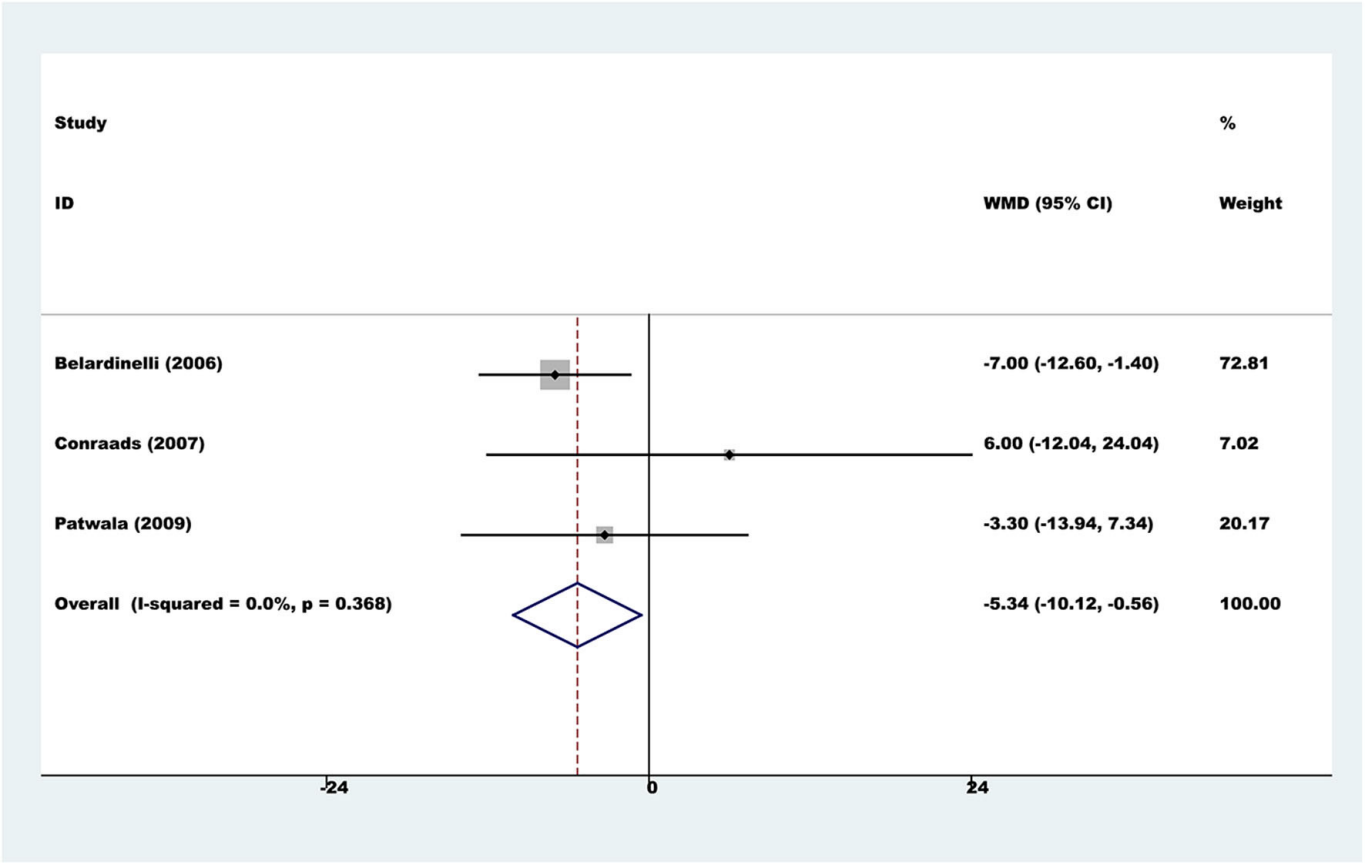
Meta-analyses of health-related quality of life.

Six trials used a variety of assessment scales to assess HRQOL. Considering the heterogeneity of different assessment scales, we did not conduct a meta-analysis across the various HRQOL measures. However, in the subgroup of three comparisons reporting the total score on the Minnesota Living with Heart Failure Questionnaire, the results showed that the exercise rehabilitation group had a higher HRQOL than the control group (fixed-effect WMD = −5.34, 95% CI −10.12 to −0.56; *P* = 0.028; *I*^2^ = 0%, Figure 5).

### Quality Assessment

We used the Cochrane risk of bias assessment tool to assess the quality of the included studies (Supplementary Images 2, 3). A number of trials failed to provide enough details to evaluate their potential risk of bias thoroughly. Two studies reported random sequence generation while three studies performed allocation concealment assessment. Two studies showed that they had taken some measures to blind those who participated in the outcome assessments. Considering the nature of interventions, blinding was not possible for participants and investigators. Four studies reported complete outcome data. Three studies were judged to have a selective reporting bias. One study had an imbalance in baseline characteristics. The overall risk of bias of the included studies was judged to be moderate. No evidence of funnel plot asymmetry was found for all-cause mortality (Egger test *P* = 0.348), serious adverse events (Egger test *P* = 0.723), peak VO_2_ (Egger test *P* = 0.453), exercise duration (Egger test *P* = 0.488), LVEF (Egger test *P* = 0.873), LVEDD (Egger test *P* = 0.157), or HRQOL (Egger test *P* = 0.104) (Supplementary Image 4).

## Discussion

This systematic review and meta-analysis identified seven RCTs with a total of 661 participants with a CRT device and compared outcomes in patients who received exercise rehabilitation and those who did not (controls). We assessed the evidence for all outcomes as low to moderate quality. The exercise rehabilitation programs consisted of all seven trials of both aerobic exercise and resistance training or stretching. The dose of exercise training ranged widely across the trials from two to five sessions per week and a duration of 15 to 60 min per session for a period of 8 to 24 weeks. The intensity of exercise in most studies was moderate, while the highest intensity was 95% of the peak heart rate. The trials had different approaches to their control group. One trial supervised the control group to maintain their regular daily routine by phone or met at the clinic every 2 weeks. The rest of the trials either did not report on this, or the control group had no limits on activities.

The meta-analysis showed that there was no evidence of a difference in all-cause mortality or serious adverse events in patients who received exercise rehabilitation and controls at the longest available follow-up. The results illustrated that exercise rehabilitation seems to be safe. However, these results must be interpreted cautiously because of the small sample sizes and short follow-up periods. Meanwhile, exercise rehabilitation had failed to decrease cardiovascular mortality and serious adverse events. Similarly, due to the risk of type II error, we take a conservative approach to the conclusions. The results are consistent with *post hoc* analyses of HF-Action, which is the largest randomized trial of exercise training in HF patients and has been conducted in patients with a right ventricular or biventricular pacemaker. The possible explanation for these results is that the significant favorable effect of exercise training on events in those without a device was abrogated by the presence of BiV pacing (Zeitler et al., 2015). The most appropriate time window is not clear; it has also been recommended that exercise training begins at least 1 week after CRT implantation (Ambrosetti et al., 2017). The interval time between CRT implantation and exercise training in the included studies reported was at least 2 weeks. Further research is needed to explore the optimal time window.

The systematic review demonstrated a potential positive effect of exercise rehabilitation on exercise capacity and cardiac function compared to controls at the end of the intervention. The observed improvements of 2.02 ml/min/kg in peak VO_2_, 102.34 s in exercise duration, and 3.89% in LVEF with exercise rehabilitation in our meta-analysis appear to have important clinical implications based on the earlier findings. Interestingly, the amelioration in LVEF was not associated with left ventricular remodeling (LVEDD WMD = −0.57 mm, 95% CI −3.20 to 2.07; *P* = 0.674). These results are in accordance with several previous studies (Conraads et al., 2007; Nobre et al., 2016; Martens et al., 2018). The mechanism of this increase is unknown. The possible explanation for these results is that the significant favorable effect of exercise training on left ventricular remodeling in those without a device was abrogated by the presence of CRT and neurohumoral blockers, which could induce significant left ventricular remodeling (Martens et al., 2018). The critical points to the subject characteristics such as the baseline of the peak VO2, LVEF, gender, etiology and medication usage might affect the study results. In the included studies, the baseline of LVEF was all <35%, and there was little difference among the studies. The baseline of the peak VO_2_, gender, etiology, and medication usage is inconsistent among studies, especially the etiology and medication usage, which could affect the response to CRT. Thus, these results must be interpreted cautiously. In this regard, further large RCTs and more stringent criteria are needed to improve the quality of studies.

The meta-analysis showed that in terms of the Minnesota Living with Heart Failure Questionnaire (MLHFQ), those who underwent exercise rehabilitation scored an average of 5.34 points lower than controls. Differences in MLHFQ scores of 5 or more have been shown to represent a significant clinical difference for patients (McAlister et al., 2004). HRQOL is also a significant outcome in RCTs involving exercise training for HF patients, as it is associated with aerobic capacity and improves meaningfully when patients with HF are undergoing exercise training (Ades et al., 2013). Thus, improving HRQOL is one of the most critical goals in the treatment of HF, although the mechanism and the interrelationship between HRQOL and prognosis are not fully understood (Luo et al., 2019).

Previous studies have shown that the clinical benefits of CRT are reduced in patients with a history of atrial fibrillation (Wilton et al., 2011; Healey et al., 2012). AF hinders atrioventricular optimization of CRT and may reduce cardiac output (Gasparini et al., 2013). Most of the included studies included different proportions of people with atrial fibrillation. However, this meta-analysis failed to further analyze the effect of heart rhythm on the synergistic effect of CRT and exercise rehabilitation. Xavier et al. reported patients in AF or sinus rhythm (SR)with a CRT device shown distinct benefits from CRT implantation and from exercise rehabilitation as an adjunctive treatment. This suggests that both mainstay and adjunctive therapy may need to be managed differently in patients with AF and SR (Melo et al., 2019). Further research is needed in the follow-up.

HF is a systemic syndrome that includes central hemodynamic changes and peripheral abnormalities. The “muscle hypothesis of cardiac failure” has been proposed to explain the peripheral abnormalities. This hypothesis proposes that inadequate skeletal muscle perfusion activates muscle ergoreceptors, leading to neurohormone activation and peripheral vasoconstriction, which stimulates the progression of the disease (Clark et al., 1996). A CRT device improves the central cardiac function, but has no significant effects on the peripheral skeletal muscle, except for muscle sympathetic nerve activity (Kuniyoshi et al., 2014). In contrast, exercise training significantly improves exercise duration, peak VO_2_, cardiac function, and HRQOL, as well as skeletal muscle function, which depends on the frequency and duration of the training program. The possible explanation behind these improvements was that CRT alone improves functional capacity and HRQOL by enhancing cardiac function, and the addition of exercise training significantly enhances the benefits by improving both the central cardiac function and the peripheral skeletal muscle function (Patwala et al., 2009).

However, CR is a comprehensive management program including patient education, nutrition consultation, close monitoring, lifestyle guidelines, and psychosocial support (Price et al., 2016). Additionally, telemonitoring of CRT devices is becoming increasingly important in long-term CR programs to adjust exercise prescription and improve adherence timely (Iliou et al., 2016). Further evidence is needed to standardize CR programs for patients with CRT.

To the best of our knowledge, this study is the most comprehensive assessment of the efficacy and safety of exercise rehabilitation in high-risk patients with HF and a CRT device. The main findings of this study suggest that exercise training can significantly improve exercise capacity and heart function in these patients. Furthermore, exercise training was associated with a higher HRQOL on follow-up.

Thus, these findings demonstrated that exercise rehabilitation is safe and effective in HF patients with CRT. This supports the wider use of exercise training in HF patients with CRT, although the development of standardized programs is necessary in more rigorous studies.

### Limitations

Our study was not without limitations. First, since there are relatively few studies on this issue, we only included seven studies in this meta-analysis. Most trials were relatively small and reported few clinical events. Second, the possibility of selection bias cannot be ruled out as only published trials were included in the meta-analysis. Third, a longer intervention period might affect outcomes, as the follow-up duration and exercise program duration might be too short for clinical outcomes. Fourth, although the trials had different approaches to the control groups, we grouped the control groups into a single control group which may have been too much of a simplification. Fifth, the intensity and duration of the exercise training in the included studies varied, and we grouped all interventions into a single intervention group, which may have also been too much of a simplification. Sixth, several included studies had a high dropout rate which may have affected the results.

## Conclusions

Our study findings suggest that exercise rehabilitation could significantly improve exercise capacity and heart function in HF patients with a CRT device. Furthermore, exercise rehabilitation was associated with a higher HRQOL on follow-up. These findings support a broader application of exercise rehabilitation among HF patients with a CRT device. Future studies are needed to assess the effects of exercise training on long-term clinical outcomes among HF patients with a CRT device.

## Data Availability Statement

All datasets generated for this study are included in the article/Supplementary Material.

## Author Contributions

LY contributed to the acquisition of data and drafting of the manuscript. SW contributed to the acquisition of data, analysis, and interpretation of data. LW contributed to the study design and obtained funding. All authors contributed to the article and approved the submitted version.

## Conflict of Interest

The authors declare that the research was conducted in the absence of any commercial or financial relationships that could be construed as a potential conflict of interest.

## Supplementary Material

Supplementary Image 1 Galbraith plot of peak oxygen uptake.

Supplementary Image 2 Risk of bias graph: review authors' judgements about each risk of bias item presented as percentages across all included studies.

Supplementary Image 3 Risk of bias summary: review authors' judgements about each risk of bias item for each included study.

Supplementary Image 4 Begg's funnel plot of publication bias. **(A)** All-cause mortality; **(B)** serious adverse events; **(C)** peak oxygen uptake; **(D)** exercise duration; **(E)** left ventricular ejection fraction; **(F)** left ventricular end diastolic dimension; **(G)** health-related quality of life.

Supplementary Table 1 Search strategy used in the present meta-analysis.
