# Supplementary figures and images for: Efficacy and Safety of Exercise Rehabilitation for Heart Failure Patients With Cardiac Resynchronization Therapy: A Systematic Review and Meta-Analysis
Source: Front Physiol. 2020 Aug 21;11:980. doi: 10.3389/fphys.2020.00980 (PMC7472379; doi:10.3389/fphys.2020.00980)

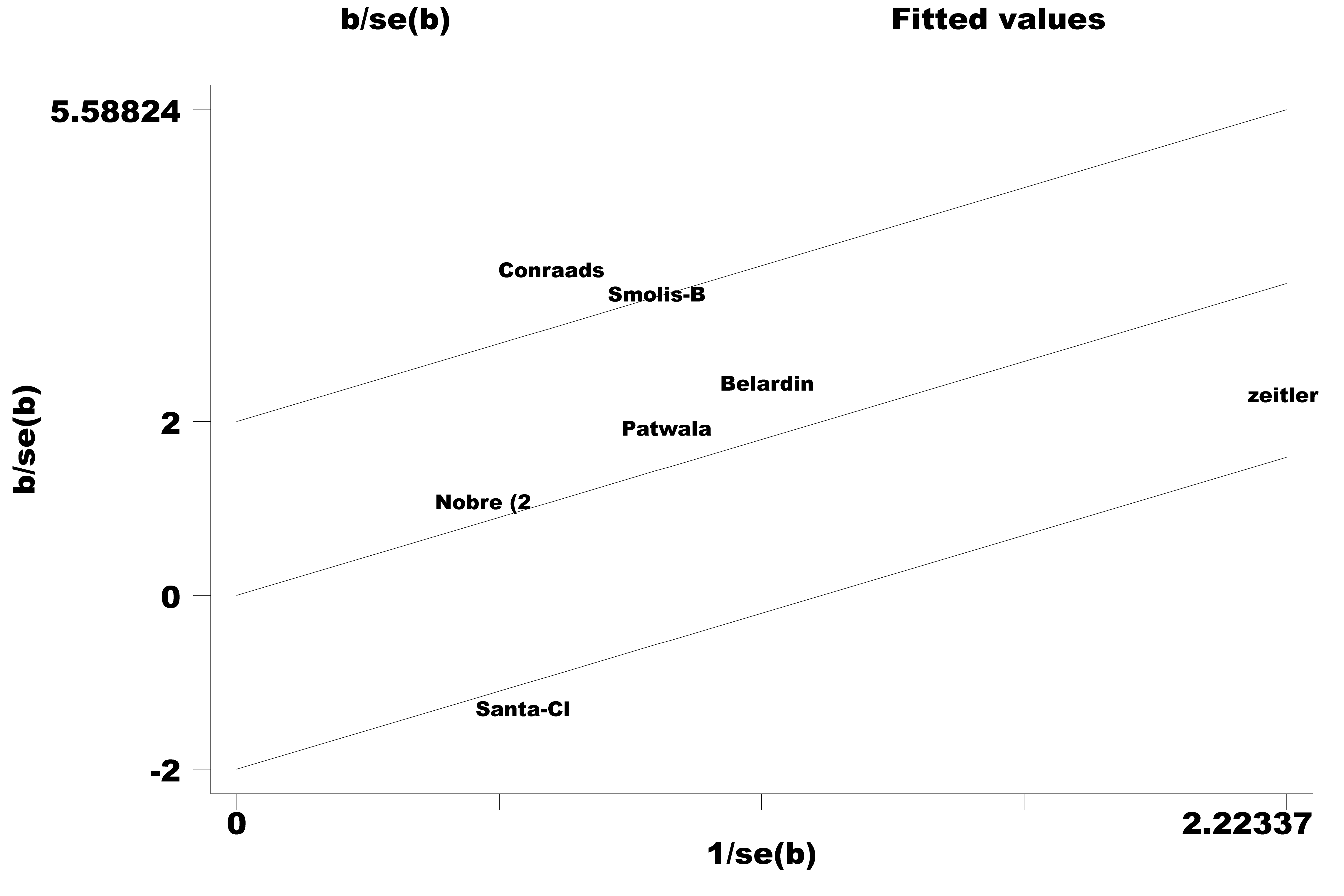

Supplement: Supplementary Image 1 — Galbraith plot of peak oxygen uptake. [file Image_1.TIF]

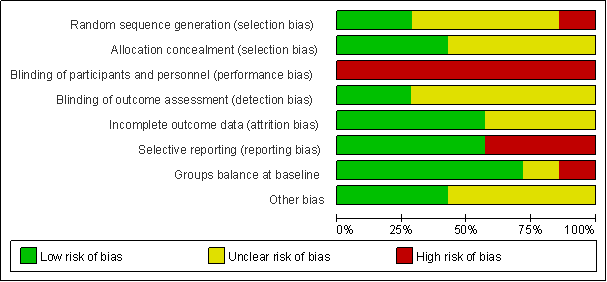

Supplement: Supplementary Image 2 — Risk of bias graph: review authors' judgements about each risk of bias item presented as percentages across all included studies. [file Image_2.TIF]

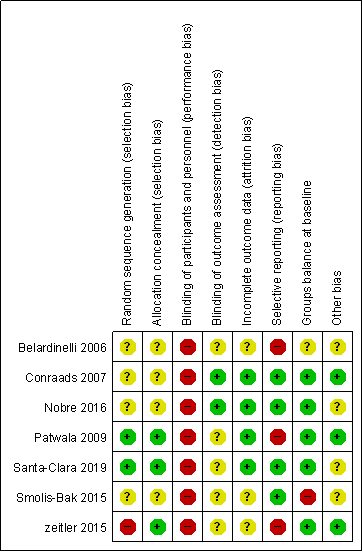

Supplement: Supplementary Image 3 — Risk of bias summary: review authors' judgements about each risk of bias item for each included study. [file Image_3.TIF]

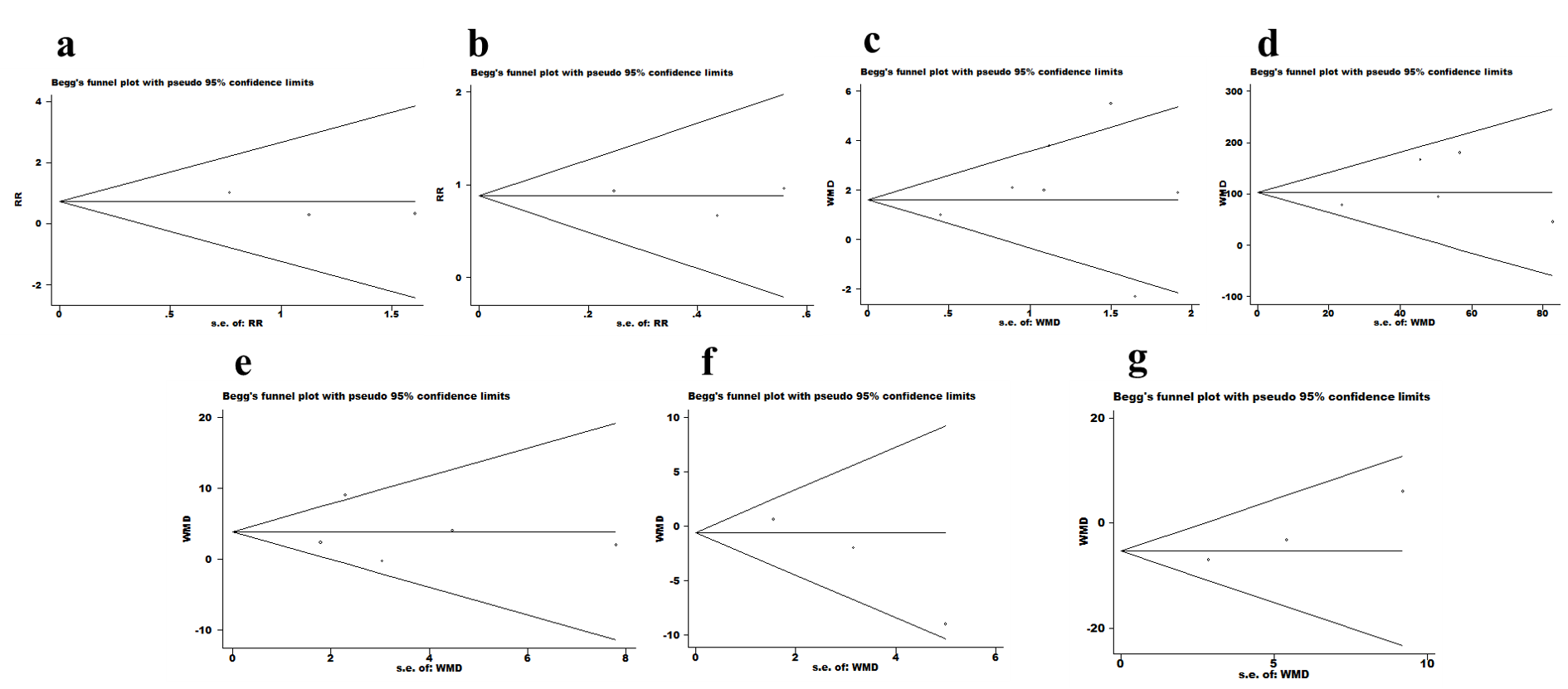

Supplement: Supplementary Image 4 — Begg's funnel plot of publication bias. (A) All-cause mortality; (B) serious adverse events; (C) peak oxygen uptake; (D) exercise duration; (E) left ventricular ejection fraction; (F) left ventricular end diastolic dimension; (G) health-related quality of life. [file Image_4.TIF]
