# Supplementary material for: Efficacy and Safety of Exercise Rehabilitation for Heart Failure Patients With Cardiac Resynchronization Therapy: A Systematic Review and Meta-Analysis
Source: Front Physiol. 2020 Aug 21;11:980. doi: 10.3389/fphys.2020.00980 (PMC7472379; doi:10.3389/fphys.2020.00980)
Supplement: Supplementary Table 1 — Search strategy used in the present meta-analysis. [file Table_1.DOCX]

**Table S1. Search strategy used in the present meta-analysis**

("Sports"[MeSH] OR "Running"[MeSH] OR "Endurance training"[MeSH] OR "Resistance training"[MeSH] OR "Walking"[MeSH] OR "Jogging"[MeSH] OR "Exercise"[MeSH] OR "Physical Exertion"[MeSH] OR "Physical Conditioning, Human"[MeSH] OR "Physical Fitness"[MeSH] OR "Rehabilitation"[MeSH] OR "rehabilitation"[All Fields] OR "sport"[All Fields] OR "sports"[All Fields] OR "exercise"[All Fields] OR "exercised"[All Fields] OR "exercising"[All Fields] OR "training"[All Fields] OR "aerobics"[all fields] OR "exertion"[All Fields] OR "physical conditioning"[all fields] OR "physical fitness"[all fields] OR "workout"[all fields] OR "working out"[all fields] OR "Running"[all fields] OR "Walking"[All Fields] OR "Jogging"[All Fields] OR "aerobic conditioning"[all fields] OR "crossfit"[All Fields]) AND ("cardiac resynchronization therapy"[MeSH] OR "resynchronization"[All Fields] OR "biventricular"[All Fields]) AND ("heart failure"[MeSH] OR "heart failure"[All Fields])
